# A Robust Diffusion Estimation Algorithm with Self-Adjusting Step-Size in WSNs

**DOI:** 10.3390/s17040824

**Published:** 2017-04-10

**Authors:** Xiaodan Shao, Feng Chen, Qing Ye, Shukai Duan

**Affiliations:** 1 College of Electronic and Information Engineering, School of Mathematics and Statistics, Southwest University, and Key Laboratory of Nonlinear Circuits and Intelligent Information Processing, Chongqing 400715, China; xiaodanshao3@126.com; 2 College of Electronic and Information Engineering, Southwest University, Chongqing 400715, China; qingye6@126.com (Q.Y.); duansk@swu.edu.cn (S.D.)

**Keywords:** robust diffusion estimation, self-adjusting step-size, non-Gaussian noise, wireless sensor networks

## Abstract

In wireless sensor networks (WSNs), each sensor node can estimate the global parameter from the local data in a distributed manner. This paper proposed a robust diffusion estimation algorithm based on a minimum error entropy criterion with a self-adjusting step-size, which are referred to as the diffusion MEE-SAS (DMEE-SAS) algorithm. The DMEE-SAS algorithm has a fast speed of convergence and is robust against non-Gaussian noise in the measurements. The detailed performance analysis of the DMEE-SAS algorithm is performed. By combining the DMEE-SAS algorithm with the diffusion minimum error entropy (DMEE) algorithm, an Improving DMEE-SAS algorithm is proposed for a non-stationary environment where tracking is very important. The Improving DMEE-SAS algorithm can avoid insensitivity of the DMEE-SAS algorithm due to the small effective step-size near the optimal estimator and obtain a fast convergence speed. Numerical simulations are given to verify the effectiveness and advantages of these proposed algorithms.

## 1. Introduction

The problem of parameter estimation, which is the indirect determination of the unknown parameters from measurements of other quantities [1,2,3,4,5,6], is a key issue in the signal processing field. Distributed estimation has become very popular for parameter estimation in wireless sensor networks. The objective is to enable the nodes to estimate a vector of parameters of interest in a distributed manner from the observed data. Distributed estimation schemes over adaptive networks can be mainly classified into incremental strategies [7,8,9], consensus strategies [10,11], and diffusion strategies [12,13,14,15,16,17,18,19,20,21,22]. In the incremental strategies, data is processed in a cyclic fashion through the network. The consensus strategies rely on the fusion of intermediate estimates of multiple neighboring nodes. In the Diffusion strategies, information is processed at all nodes while the nodes communicate with all their neighbors to share their intermediate estimates. The diffusion strategies are particularly attractive because they are robust, flexible and fully-distributed, such as the diffusion least mean squares (DLMS) algorithm [12]. In this paper, we focus on the diffusion estimation strategies.

The performance of distributed estimation degrades severely when the signals are perturbed by non-Gaussian noise. Non-Gaussian noise may be natural, due to atmospheric phenomena, or man-made, due to either electric machinery present in the operation environment, or multipath telecommunications signals [23,24,25]. Recently, some researchers focus on improving robustness for non-Gaussian noise of distributed estimation methods. The efforts are mainly directed at searching for a more robust cost function to replace the MSE criterion, which is optimal only when the measurement noise is Gaussian. To address this problem, the diffusion least mean *p*-power (DLMP) based on *p*-norm error criterion was proposed to estimate the parameters of the wireless sensor networks [26]. The correntropy as a nonlinear similarity measure has been successfully used as a robust and efficient cost function for non-Gaussian signal processing [27,28,29,30]. In [27], two robust MCC based diffusion algorithms, namely the Adapt-then-Combine (ATC) and Combine-then-Adapt (CTA) diffusion maximum correntropy criterion (DMCC) algorithms, are developed to improve the performance of the distributed estimation over network in impulsive noise environments.

The error entropy criterion based on the minimum error entropy (MEE) method also has shown its ability to achieve more accurate estimates than mean-square error (MSE) under non-Gaussian noise [31,32,33,34,35,36,37]. In [31], the diffusion minimum error entropy (DMEE) was proposed. The DMEE algorithm achieved improved performance for non-Gaussian noise with the fixed step-size, but it still suffers from conflicting requirements between convergence rate and the steady-state mean square error. A large step-size leads to a fast convergence rate but a large mean-square error at the steady state. For this problem, variable step-size techniques have been widely used to improve the convergence of diffusion LMS algorithms remarkably by adjusting the step-size appropriately [38,39,40,41]. Lee et al. [38] proposed a novel variable step-size diffusion LMS algorithm which controls the step-size suboptimally to attain the minimum mean square error at each time instant. In [41], Abdolee investigated the effect of adaptation step-sizes on the tracking performance of DLMS algorithms in networks under non-stationary signal conditions. However, to the best of our knowledge, the variable step-size technique has not been extended to the field of distributed minimum error entropy estimation for non-Gaussian noise yet.

In this paper, we incorporate the minimum error entropy criterion with self-adjusting step-size (MEE-SAS) [42] into the cost function in diffusion distributed estimation. Then, we figure out the diffusion-strategy solutions, which are referred to as the diffusion MEE-SAS (DMEE-SAS) algorithm. Numerical simulation results show that the DMEE-SAS algorithm outperforms DLMS, DLMP and DMEE algorithms when the noise is modeled to be non-Gaussian noise. We also design an Improving DMEE-SAS algorithm by using a switching scheme between DMEE-SAS and DMEE algorithms for a non-stationary environment, which tracks the changing estimator very effectively. The Improving DMEE-SAS algorithm can avoid the small effective step-size of the DMEE-SAS algorithm when it is close to the optimal estimator.

We organize the paper as follows. In Section 2, we briefly revisit the minimization error entropy criterion. In Section 3, firstly, we propose the DMEE-SAS algorithm and analyze the mean, mean square and instantaneous MSD performance for the DMEE-SAS algorithm. Then, we propose the Improving DMEE-SAS algorithm for a non-stationary scenario. Simulation results are shown in Section 4. Finally, we draw conclusions in Section 5.

## 2. Minimization Error Entropy Criterion

Considering the limited computational capability and limited memory space for nodes in real distributed networks, this paper is based on an MEE criterion, which is simple enough and has good estimation accuracy. Important properties of MEE can be found in [32,35,37]. In many real world applications, the MEE estimator can outperform significantly the well-known MSE estimator and show strong robustness to noises, especially when data are contaminated by non-Gaussian noises. In this subsection, we introduce an MEE criterion, which could be used to derive a robust diffusion estimation algorithm with a self-adjusting step-size (DMEE-SAS) algorithm.

The aim of the adaptive signal processing problem is to minimize the difference between the desired and the system outputs, which is defined as error *e*. For the evaluation of the error entropy, we seek to estimate entropy directly from the error samples. Therefore, system parameters can be estimated by minimizing the *Renyi’s* entropy of the error *e*. *Renyi’s* entropy is given by

(1) $$H_{\alpha }\left(e\right)=\frac{1}{1‒\alpha }log\int q^{\alpha }\left(e\right)de,$$

where $q^{\alpha }\left(e\right)$ is the probability density function of a continuous error *e*, and α is a parameter. When parameter α is set as 2, Equation (1) is quadratic *Renyi’s* entropy. Using a Gaussian kernel with kernel size σ, we can obtain a convenient evaluation of the integral operator in the formulation of quadratic *Renyi’s* entropy as follows:

(2) $$\begin{array}{cc} H_{2}\left(e\right) & =‒log\int q^{2}\left(e\right)de\\ & =‒log\int {\left(\frac{1}{N}\sum_{i=1}^{N}G_{\sigma }\left(e‒e_{i}\right)\right)}^{2}de\\ & =‒\log(V(e)), \end{array}$$

where $e=[e_{1},e_{2},\cdots ,e_{N}]$ is *N* independent and identically distributed samples, and the Gaussian kernel is defined as

$$G_{\sigma \sqrt{2}}\left(e‒e_{i}\right)=\frac{1}{\sigma \sqrt{2\pi }}\exp\left(‒\frac{1}{2\sigma^{2}}{\left(e‒e_{i}\right)}^{2}\right).$$

The information V(e) is quadratic information potential and is defined as the expectation of probability density function, $V\left(e\right)=E\left[q\left(e\right)\right]$. The quadratic information potential V(e) can be easily estimated by using a simple and effective nonparametric estimator

(3) $$V\left(e\right)=\frac{1}{N^{2}}\sum_{i=1}^{N}\sum_{j=1}^{N}G_{\sigma \sqrt{2}}\left(e_{j}‒e_{i}\right)\leq V\left(0\right)\;\;=\frac{1}{\sigma \sqrt{2\pi }}.$$

The maximum value of the quadratic information potential V(0) will be achieved when $e_{1}=e_{2}=\cdots =e_{N}$. The above results are obtained in the case of batch mode, where the *N* data points are fixed. For online training methods, in order to reduce calculation costs, the estimate of quadratic information potential can be approximated stochastically by dropping the time average in (3), leading to

(4) $$V\left(e_{i}\right)\approx \frac{1}{L}\sum_{j=i‒L+1}^{i}G_{\sigma \sqrt{2}}\left(e_{i}‒e_{j}\right),$$

where *L* is the latest *L* samples at time *i*.

Obviously, to minimize the error entropy is equivalent to maximizing the quadratic information potential since the log is a monotonic function. Therefore, the cost function for the MEE criterion is given by

(5) $$J_{MEE}\left(e\right)=maxV\left(e\right).$$

The selection of the kernel size σ is an important step in estimating the information potential and is critical to the success of information theoretic criteria. In particular, increasing the kernel size leads to a stretching effect on the performance surface in the weight space, which results in increased accuracy of the quadratic approximation around the optimal point [43]. In order to ensure accuracy, in the following, a large enough kernel size can be used during the adaptation process, which is commonly used in information theoretic criteria [42,44].

## 3. Proposed Algorithms

As mentioned in the Introduction, the diffusion minimum error entropy algorithm achieved improved performance for non-Gaussian noise with the fixed step-size, but it still suffers from conflicting requirements between convergence rate and the steady-state mean square error. Therefore, we consider a new cost function, which can achieve fast convergence speed and strong robustness against non-Gaussian noise.

### 3.1. Diffusion MEE-SAS Algorithm

Consider a connected wireless sensor networks with *K* nodes. k∈{1,2,…,K} is the node index and *i* is the time index. To proceed with the analysis, we assume a liner measurement model as follows:

(6) $$d_{k,i}=u_{{}_{k,i}}^{T}w^{0}+v_{k,i},$$

where $w^{0}$ is a M×1 deterministic but unknown vector, $d_{k,i}$ is a scalar measurement of some random process, $u_{k,i}$ is the M×1 regression vector at time *i* with zero mean, and $v_{k,i}$ is the random noise signal at time *i* with zero mean. For each node *k*, we have

(7) $$V\left(e_{k}\right)=\frac{1}{N^{2}}\sum_{i=1}^{N}\sum_{j=1}^{N}G_{\sigma \sqrt{2}}\left(e_{k,j}‒e_{k,i}\right)\leq V\left(0\right)\;\;=\frac{1}{\sigma \sqrt{2\pi }},$$

where $e_{k,i}=d_{k,i}‒u_{k,i}^{T}w$. The maximum value V(0) will be achieved when $e_{k,i}=e_{k,j},\;\;j=i‒L+1,i‒L+2,\cdots ,i$.

We seek an estimate of $w^{0}$ by minimizing a linear combination of local information. As explained in Section 2, minimizing a linear combination of the local information is equivalent to maximizing a linear combination of the local quadratic information potential $V(e_{k,i})$. To maximize the information potential is equivalent to minimizing the following cost function:

(8) $$\begin{array}{cc} J_{k}\left(w\right) & =\sum_{l\in N_{k}}c_{l,k}{\left[V\left(0\right)‒V\left(e_{l}\right)\right]}^{2}\\ & =\sum_{l\in N_{k}}F_{l}\left(w\right), \end{array}$$

where

$$F_{l}\left(w\right)={\left[V\left(0\right)‒V\left(e_{l}\right)\right]}^{2}.$$

$N_{k}$ denotes the one-hop neighbor set of node *k*, and $\left\{c_{lk}\right\}$ are some non-negative cooperative coefficients satisfying $c_{lk}=0$ if $l\notin N_{k}$, $1_{N}^{T}\bar{C}=1_{N}^{T}$ and $\bar{C}1_{N}=1_{N}$. Here, $\bar{C}$ is a N×N matrix with individual entries $\left\{c_{lk}\right\}$ and $1_{N}$ is a N×1 all-unity vector. The gradient of the individual local cost function is given by

(9) $$\nabla J_{k}\left(w\right)=\sum_{l\in N_{k}}c_{lk}f_{l}\left(w\right),$$

where

(10) $$f_{l}\left(w\right)=\left(\frac{2}{\sigma^{2}N^{2}}\right)\left(V\left(0\right)‒V\left(e_{k}\right)\right)\sum_{i=1}^{N}\sum_{j=1}^{N}G_{\sigma \sqrt{2}}\left(e_{k,j}‒e_{k,i}\right)\left(e_{k,j}‒e_{k,i}\right)\left(u_{k,i}‒u_{k,j}\right).$$

We can replace the estimate of quadratic information potential by the stochastic quadratic information potential, leading to

(11) $$\nabla {\hat{J}}_{k}\left(w\right)=\sum_{l\in N_{k}}c_{lk}{\hat{f}}_{l}\left(w\right),$$

where

(12) $${\hat{f}}_{l}\left(w\right)=\left(\frac{2}{\sigma^{2}L}\right)\left(V\left(0\right)‒V\left(e_{k,i}\right)\right)\sum_{j=i‒L+1}^{i}G_{\sigma \sqrt{2}}\left(e_{k,i}‒e_{k,j}\right)\left(e_{k,i}‒e_{k,j}\right)\left(u_{k,j}‒u_{k,i}\right),$$

where

(13) $$V\left(e_{l,i}\right)\approx \frac{1}{L}\sum_{j=i‒L+1}^{i}G_{\sigma \sqrt{2}}\left(e_{l,i}‒e_{l,j}\right).$$

Iterative steepest-descent solution for estimating $w^{0}$ at each node *k* can thus be derived as

(14) $$\begin{array}{cc} w_{k,i+1} & =w_{k,i}‒\mu_{k}\nabla {\hat{J}}_{k}\left(w\right)\\ & =w_{k,i}‒\mu_{k}\frac{2}{\sigma^{2}L}\sum_{l\in N_{k}}c_{lk}\left[V\left(0\right)‒V\left(e_{l,i}\right)\right]\sum_{j=i‒L+1}^{i}G_{\sigma \sqrt{2}}\left(e_{l,i}‒e_{l,j}\right)\left(e_{l,i}‒e_{l,j}\right)\left(u_{l,j}‒u_{l,i}\right), \end{array}$$

where $\mu_{k}$ is a positive step size. Using the general framework for diffusion-based distributed adaptive optimization [13], an adapt-then-combine (ATC) strategy for diffusion MEE-SAS algorithm can be formulated as

(15) $$\left\{\begin{array}{c} \varphi_{k,i+1}=w_{k,i}‒\mu_{k}\frac{2}{\sigma^{2}L}\left[V\left(0\right)‒V\left(e_{k,i}\right)\right]\sum_{j=i‒L+1}^{i}G_{\sigma \sqrt{2}}\left(e_{k,i}‒e_{k,j}\right)\left(e_{k,i}‒e_{k,j}\right)\left(u_{k,j}‒u_{k,i}\right),\\ w_{k,i+1}=\sum_{l\in N_{k}}c_{lk}\varphi_{l,i+1}. \end{array}\right.$$

According to Equation (15), the DMEE-SAS algorithm can be seen as a diffusion estimation algorithm with variable step size $\mu_{k}\left(i\right)$, where

(16) $$\mu_{k}\left(i\right)=2\mu_{k}\left[V\left(0\right)‒V\left(e_{k,i}\right)\right].$$

The DMEE-SAS algorithm is described formally in Algorithm 1.
**Algorithm 1:** DMEE-SAS Algorithm **Initialize:**
$w_{k,i}=0$ **for**
i=1:T **for each node**
*k*:  **Adaptation**  $\mu_{k}\left(i\right)=2\mu_{k}\left[V\left(0\right)‒V\left(e_{k,i}\right)\right]$  $\varphi_{k,i+1}=w_{k,i}‒\mu_{k}\left(i\right)\frac{1}{\sigma^{2}L}\sum_{j=i‒L+1}^{i}G_{\sigma \sqrt{2}}\left(e_{k,i}‒e_{k,j}\right)\left(e_{k,i}‒e_{k,j}\right)\left(u_{k,j}‒u_{k,i}\right)$  **Combination**  $w_{k,i+1}=\sum_{l\in N_{k}}c_{lk}\varphi_{l,i+1}$ **end for**

In the adaption step of DMEE-SAS algorithm, $V\left(0\right)‒V\left(e_{k,i}\right)$ is close to V(0) when the algorithm starts, and it is close to 0 when the algorithm begins to converge. $V\left(0\right)‒V\left(e_{k,i}\right)$ is always a non-negative scalar quantity, which can accelerate the rate of convergence and achieve small steady-state estimation errors. The fast convergence rate and the small steady-state estimation errors of the DMEE-SAS algorithm can be established against non-Gaussian noise in the measurements.

### 3.2. Performance Analysis

In this section, we analyze the mean, mean-square and instantaneous MSD performance of the DMEE-SAS algorithm. For tractability of the analysis, here we focus on the case of batch mode. To briefly present the convergence property of the proposed algorithm in terms of global quantities, the following notations are introduced: $M=diag\{\mu_{1}I_{M},\ldots ,\mu_{K}I_{M}\}$, $W_{i}=col\left\{w_{1,i},\cdots w_{K,i}\right\}$, $w^{\left(0\right)}=col\left\{w^{0},\cdots ,w^{0}\right\}$, ${\tilde{W}}_{i}=col\left\{{\tilde{w}}_{1,i}\cdots {\tilde{w}}_{K,i}\right\}$, $S=col\{s_{1}\left(w^{0}\right),\cdots ,s_{K}\left(w^{0}\right)\}$, $C={\bar{C}}^{T}⊗I_{M}$, $I_{M}$ is the identity matrix.

In order to make the analysis tractable, the followings are assumed:

Assumption 1: The regressor $u_{k,i}$ is independent identically distributed (i.i.d) in time and spatially independent, and $E[u_{k,i}]=0$, $R_{k}=E\left[u_{k,i}^{T}u_{k,i}\right]$.

Assumption 2: The input noise $v_{k,i}$ is super-Gaussian noise. In addition, $v_{k,i}$ and the regressor $u_{k,i}$ are independent from each other. We have $E[v_{k,i}]=0$ and $E\left[v_{k,i}^{2}\right]=\xi_{k}$.

Assumption 3: The step-sizes, $\mu_{k}$, ∀k, are small enough such that their squared values are negligible.

#### 3.2.1. Mean Performance

Because the input signal and output noises are generated from stationary and ergodic processes, the double time average in Equation (10) can be replaced by the expectation, leading to

(17) $$f_{k}\left(w\right)\approx \frac{2}{\sigma^{2}}\left(V\left(0\right)‒E\left[G_{\sigma \sqrt{2}}\left(e_{k,j}‒e_{k,i}\right)\right]\right)E\left[G_{\sigma \sqrt{2}}\left(e_{k,j}‒e_{k,i}\right)\left(e_{k,j}‒e_{k,i}\right)\left(u_{k,i}‒u_{k,j}\right)\right].$$

We consider the gradient error caused by approximating the quadratic information potential $V(e_{k,i})$ with their instantaneous values [45]. The gradient error at iteration *i* and each node *k* is defined as follows:

(18) $$s_{k}\left(w_{k,i}\right)={\hat{f}}_{k}\left(w_{k,i}\right)‒f_{k}\left(w_{k,i}\right).$$

Using Equation (15), the update equation of the intermediate estimate can be rewritten as

(19) $$\varphi_{k,i+1}=w_{k,i}‒\mu_{k}\left(f_{k}\left(w_{k,i}\right)+s_{k}\left(w_{k,i}\right)\right).$$

According to [44], when input signal-to-noise ratio is not too low, the error should be small on the whole. Therefore, for a relative large kernal size σ, when $w=w^{0}$, ($(e_{k,i}‒e_{k,j})/\sigma \approx 0$ and $G_{\sigma \sqrt{2}}\left(e_{k,i}‒e_{k,j}\right)\approx \frac{1}{\sigma \sqrt{2\pi }}$. Therefore, the Hessian matrix function $H_{k}\left(w^{0}\right)$ of $F_{l}\left(w\right)$ is calculated as:

(20) $$\begin{array}{cc} H_{k}\left(w^{0}\right) & =\frac{\partial f_{k}\left(w\right)}{\partial w}{|}_{w^{0}}\\ & =\frac{\partial }{\partial w}\frac{2}{\sigma^{2}}\left(V\left(0\right)‒E\left[G_{\sigma \sqrt{2}}\left(e_{k,j}‒e_{k,i}\right)\right]\right)E\left[G_{\sigma \sqrt{2}}\left(e_{k,j}‒e_{k,i}\right)\left(e_{k,j}‒e_{k,i}\right)\left(u_{k,i}‒u_{k,j}\right)\right]\\ & =\frac{2}{\sigma^{2}}\left(V\left(0\right)‒E\left[G_{\sigma \sqrt{2}}\left(e_{k,j}‒e_{k,i}\right)\right]\right)E[G_{\sigma \sqrt{2}}\left(e_{k,j}‒e_{k,i}\right){\left(u_{k,i}‒u_{k,j}\right)}^{T}\left(u_{k,i}‒u_{k,j}\right)‒\\ & \;\;\;\frac{1}{\sigma^{2}}G_{\sigma \sqrt{2}}\left(e_{k,j}‒e_{k,i}\right){\left(e_{k,j}‒e_{k,i}\right)}^{2}{\left(u_{k,i}‒u_{k,j}\right)}^{T}\left(u_{k,i}‒u_{k,j}\right)]\\ & \;\;\;+\frac{2}{\sigma^{4}}E{\left[G_{\sigma \sqrt{2}}\left(e_{k,j}‒e_{k,i}\right)\left(e_{k,j}‒e_{k,i}\right)\left(u_{k,i}‒u_{k,j}\right)\right]}^{T}E\left[G_{\sigma \sqrt{2}}\left(e_{k,j}‒e_{k,i}\right)\left(e_{k,j}‒e_{k,i}\right)\left(u_{k,i}‒u_{k,j}\right)\right]\\ & =\frac{1}{\sigma^{6}\pi }E\left[v_{{}_{k,i}}^{2}+v_{{}_{k,j}}^{2}\right]E\left[u_{k,i}^{T}u_{k,i}+u_{k,j}^{T}u_{k,j}\right]\\ & =\;\frac{4\xi_{k}R_{k}}{\sigma^{6}\pi }. \end{array}$$

Based on the Theorem 1.2.1 of [46], we obtain

(21) $$\begin{array}{cc} f_{k}\left(w_{k,i}\right) & =f_{k}\left(w^{0}\right)‒\left(\int_{0}^{1}H_{k}\left(w^{0}‒x{\tilde{w}}_{k,i}\right)dx\right){\tilde{w}}_{k,i}\\ & =‒\left(\int_{0}^{1}H_{k}\left(w^{0}‒x{\tilde{w}}_{k,i}\right)dx\right){\tilde{w}}_{k,i}, \end{array}$$

where ${\tilde{w}}_{k,i}=w^{0}‒w_{k,i}$ is the weight error vector for node *k*. We assume that the estimate of each node converges to the vicinity of the unknown vector $w^{0}$. Therefore, ${\tilde{w}}_{k,i}$ is small enough such that it is negligible, yielding

(22) $$\begin{array}{cc} f_{k}\left(w_{k,i}\right) & \approx ‒\left(\int_{0}^{1}H_{k}\left(w^{0}\right)dx\right){\tilde{w}}_{k,i}\;\\ & =‒H_{k}\left(w^{0}\right){\tilde{w}}_{k,i}. \end{array}$$

We can also obtain the approximation of the gradient error at the vicinity of $w^{0}$, which is given by

(23) $$\begin{array}{cc} s_{k}\left(w_{k,i}\right) & \approx s_{k}\left(w^{0}\right)\\ & ={\hat{f}}_{k}\left(w^{0}\right)‒f_{k}\left(w^{0}\right)\\ & =\frac{2}{\sigma^{2}L}\left(V\left(0\right)‒\frac{1}{L}\sum_{j=i‒L+1}^{i}G_{\sigma \sqrt{2}}\left(v_{l,i}‒v_{l,j}\right)\right)\sum_{j=i‒L+1}^{i}\\ & \;\;G_{\sigma \sqrt{2}}\left(v_{k,i}‒v_{k,j}\right)\left(v_{k,i}‒v_{k,j}\right)\left(u_{k,j}‒u_{k,i}\right). \end{array}$$

Substituting Equations (22) and (23) into Equation (19), an approximation of intermediate estimate can be obtained at the vicinity of

(24) $$\varphi_{k,i+1}=w_{k,i}+\mu_{k}\left(H_{k}\left(w^{0}\right){\tilde{w}}_{k,i}‒s_{k}\left(w^{0}\right)\right).$$

By substituting Equation (24) into the second equation of Equation (15), we get the estimate of unknown parameter as follows:

(25) $$w_{k,i+1}=\sum_{l\in N_{k}}c_{lk}\left[w_{l,i}+\mu_{k}\left(H_{k}\left(w^{0}\right){\tilde{w}}_{l,i}‒s_{l}\left(w^{0}\right)\right)\right].$$

Using global quantities defined above gives the update equation for the network estimate vector as

(26) $$W_{i+1}=C\left(W_{i}+MH{\tilde{W}}_{i}‒MS\right),$$

where *H* collects the Hessian matrix across the network into the global vector $H=diag(H_{1}\left(w^{0}\right),\cdots ,H_{N}\left(w^{0}\right))$. Noting that $Cw^{\left(0\right)}=w^{\left(0\right)}$, subtraction of both sides of Equation (26) from $w^{\left(0\right)}$ gives

(27) $${\tilde{W}}_{i+1}=C\left(I_{MN}‒MH\right){\tilde{W}}_{i}+CMS.$$

In view of assumptions A1 and A2, ${\tilde{W}}_{i}$, *H* and *C* are independent of each other. Hence, taking expectation of both sides of Equation (27) leads to

(28) $$E\left[{\tilde{W}}_{i+1}\right]=E\left[C\right]\left(I_{MN}‒MH\right)E\left[{\tilde{W}}_{i}\right]+CME\left[S\right].$$

We can easily find that $E\left[S\right]=col\{E\left[s_{1}\left(w^{0}\right),\cdots ,s_{N}\left(w^{0}\right)\right]\}=0$, and Equation (28) has therefore been reduced to this form

(29) $$E\left[{\tilde{W}}_{i+1}\right]=E\left[C\right]\left(I_{MN}‒MH\right)E\left[{\tilde{W}}_{i}\right].$$

From Equation (29), we observe that, in order to be stable for Algorithm 1 in the mean sense, the matrix $E\left[C\right]\left(I_{MN}‒MH\right)$ should be stable. All the entries of E(C) are non-negative and all the rows of it add up to unity. Therefore, to ensure the stability in the mean, it should hold that

(30) $$\left|\lambda_{\max}\left\{I_{MN}‒MH\right\}\right|<1.$$

We use the notion $\lambda_{\max}\left(A\right)$ to denote the maximum eigenvalue of a Hermitian matrix *A*. Thus, we note that a sufficient condition for unbiasedness is

(31) $$0<\mu_{k}<\frac{2}{\lambda_{\max}\left\{H_{k}\left(w^{0}\right)\right\}}\;\;\;\;\;\;=\frac{\sigma^{6}\pi }{2\lambda_{max}\left\{R_{k}\xi_{k}\right\}}.$$

#### 3.2.2. Mean-Square Performance

In order to make the presentation clearer, we shall introduce the following notation

$$\Gamma =\left(I_{MN}‒MH\right)C^{T}\Sigma C\left(I_{MN}‒MH\right).$$

Performing weighted energy balance on both sides of Equation (27) and taking expectations gives

(32) $$E\left[{∥{\tilde{W}}_{i+1}∥}_{\Sigma }^{2}\right]=E\left[{∥{\tilde{W}}_{i}∥}_{\Gamma }^{2}\right]+E\left[S^{T}MC^{T}\Sigma CMS\right],$$

where Σ is an arbitrary symmetric nonnegative-definite matrix, and the notion ${∥a∥}_{\Sigma }^{2}=a^{T}\Sigma a$ represents a weighted vector norm for any Hermitian Σ>0. By defining

r=vec{E[Γ]},θ=vec{Σ},

where the vec(.) notation stacks the columns of its matrix argument on top of each other. We can modify Equation (32) to

(33) $$E\left[{∥{\tilde{W}}_{i+1}∥}_{\theta }^{2}\right]=E\left[{∥{\tilde{W}}_{i}∥}_{r}^{2}\right]+E\left[S^{T}MC^{T}\Sigma CMS\right].$$

Using the following relationship of the vectorization operator and the Kronecker product [47]:

$$vec\left(ABC\right)=\left(C^{T}⊗A\right)vec\left\{B\right\}.$$

We can obtain that

(34) r=ϕθ,

where

(35) $$\phi =E[\left(I_{MN}‒MH\right)⊗\left(I_{MN}‒MH\right)]\beta ,$$

$$\beta =E[C^{T}⊗C^{T}].$$

Considering Assumption 3, we can approximate Equation (35) as

(36) $$\begin{array}{cc} \phi & \approx (I_{M^{2}N^{2}}‒I_{MN}⊗MH‒MH⊗I_{MN})\beta \\ & =\left(I_{MN}‒MH\right)⊗\left(I_{MN}‒MH\right)\beta . \end{array}$$

Using the following relationship of the vectorization operator and the matrix trace [47]:

$$Tr\left\{A^{T}B\right\}=vec^{T}\left(B\right)vec\left(A\right).$$

We find that

(37) $$E\left[S^{T}MC^{T}\Sigma CMS\right]=vec^{T}\left\{Q\right\}\beta \theta ,$$

where

$$Q=E[MSS^{T}M].$$

Substituting Equations (34) and (37) into Equation (33), we can then reformulate recursion as follows:

(38) $$E\left[{∥{\tilde{W}}_{i+1}∥}_{\theta }^{2}\right]=E\left[{∥{\tilde{W}}_{i}∥}_{\phi \theta }^{2}\right]+vec^{T}\left\{Q\right\}\beta \theta .$$

It is known that Equation (38) is stable and convergent if the matrix ϕ is stable [48], form the Equation

$$\beta^{T}1_{M^{2}N^{2}}=E\left[\left(C⊗I_{M}\right)1_{MN}⊗\left(C⊗I_{M}\right)1_{MN}\right]=1_{M^{2}N^{2}},$$

We know that all the entries of β in Equation (37) are non-negative, and all its columns sum up to unity. Using the property $\lambda \left(A⊗A\right)=\lambda^{2}\left(A\right)$, the stability of ϕ has the same conditions as the stability of $I_{MN}‒MH$. Therefore, we choose the step size in accordance with Equation (31), which can keep the DMEE-SAS stable in the mean-square sense.

#### 3.2.3. Instantaneous MSD

In order to analyze instantaneous mean-square-error (MSD), we can exploit the liberty of choosing θ at time *i*. Then, Expression (38) gives:

(39) $$E\left[{∥{\tilde{W}}_{n+1}∥}_{\phi^{i‒n}\theta }^{2}\right]=E\left[{∥{\tilde{W}}_{n}∥}_{\phi^{i‒n+1}\theta }^{2}\right]+vec^{T}\left\{Q\right\}\beta \phi^{i‒n}\theta .$$

The sum of both sides of Equation (39) for n=0,1,…,i‒1 can be given by

(40) $$E\left[{∥{\tilde{W}}_{i}∥}_{\theta }^{2}\right]=E\left[{∥{\tilde{W}}_{0}∥}_{\phi^{i}\theta }^{2}\right]+vec^{T}\left\{Q\right\}\sum_{n=0}^{i‒1}\beta \phi^{n}\theta .$$

We can also adopt a similar way to describe the time instant i+1, given by

(41) $$E\left[{∥{\tilde{W}}_{i+1}∥}_{\theta }^{2}\right]=E\left[{∥{\tilde{W}}_{0}∥}_{\phi^{i+1}\theta }^{2}\right]+vec^{T}\left\{Q\right\}\sum_{n=0}^{i}\beta \phi^{n}\theta .$$

Subtraction of both sides of Equation (40) from Equation (41) gives

(42) $$E\left[{∥{\tilde{W}}_{i+1}∥}_{\theta }^{2}\right]=E\left[{∥{\tilde{W}}_{i}∥}_{\theta }^{2}\right]‒E\left[{∥{\tilde{W}}_{0}∥}_{\phi^{i}\left(I_{M^{2}N^{2}}‒\phi \right)\theta }^{2}\right]+vec^{T}\left\{Q\right\}\beta \phi^{n}\theta .$$

By setting

$$\theta =vec\left\{I_{MN}\right\}$$

in Equation (44) and dividing both sides of it by *N*, the instantaneous MSD for the whole network are computed by:

(43) $$\frac{1}{N}\sum_{k=1}^{N}E\left[{∥{\tilde{w}}_{k,i}∥}^{2}\right]=\frac{1}{N}E\left[{∥{\tilde{W}}_{i}∥}^{2}\right],$$

where ${\tilde{W}}_{i}$ can be obtained by the following iteration:

(44) $$\frac{1}{N}E\left[{∥{\tilde{W}}_{i+1}∥}^{2}\right]=\frac{1}{N}E\left[{∥{\tilde{W}}_{i}∥}^{2}\right]‒\frac{1}{N}E\left[{∥{\tilde{W}}_{0}∥}_{\phi^{i}\left(I_{M^{2}N^{2}}‒\phi \right)vec\left\{I_{MN}\right\}}^{2}\right]+\frac{1}{N}vec^{T}\left\{Q\right\}\beta \phi^{i}vec\left\{I_{MN}\right\}.$$

### 3.3. An Improving Scheme for the DMEE-SAS Algorithm

The too small effective step size near the optimal estimator will hinder the tracking ability of the DMEE-SAS algorithm in a non-stationary environment. In a non-stationary environment, the optimal estimator has small changes. A random-walk model is commonly used in the literature to describe the non-stationarity of the weight vector [48].

Therefore, we try to combine the DMEE-SAS algorithm with the DMEE algorithm [31] in a non-stationary environment where tracking is important. The DMEE-SAS algorithm should be used due to the faster convergence when the algorithm starts, and the DMEE algorithm should be used when the algorithm begins to converge. We use the Lyapunov stability theory [49] to analyze the switching time for each node.

The Lyapunov energy function is a method for analyzing the convergence characteristics of dynamic systems. The cost function can be viewed as a Lyapunov energy function. For the DMEE-SAS algorithm, the continuous-time learning rule is

(45) $$\dot{w}=‒\mu_{DMEE‒SAS}\frac{\partial J_{k}{\left(w\right)}_{DMEE‒SAS}}{\partial w}.$$

The temporal dynamics for the Lyapunov energy that describes the DMEE-SAS algorithm can be obtained as follows:

(46) $$\begin{array}{cc} {\dot{J}}_{k}{\left(w\right)}_{DMEE‒SAS} & =\sum_{l\in N_{k}}c_{lk}\left(‒2\right)\left[V\left(0\right)‒V\left(e_{l,i}\right)\right]\frac{\partial V{\left(e_{l,i}\right)}^{T}}{\partial w}\dot{w}\\ & =\sum_{l\in N_{k}}{c_{lk}\left(‒4\right)\mu_{k,DMEE‒SAS}{\left[V\left(0\right)‒V\left(e_{l,i}\right)\right]}^{2}∥\frac{\partial V(e_{l,i})}{\partial w}∥}^{2}. \end{array}$$

The individual local energy function for DMEE algorithm can be written as

(47) $$J_{k}{\left(w\right)}_{DMEE}=‒\sum_{l\in N_{k}}c_{lk}V\left(e_{l,i}\right).$$

For the DMEE algorithm, the continuous-time learning rule is

(48) $$\dot{w}=‒\mu_{DMEE}\frac{\partial J_{k}{\left(w\right)}_{DMEE}}{\partial w}.$$

In a similar way, the temporal dynamics for the Lyapunov energy that describes the DMEE algorithm can be obtained as follows:

(49) $$\begin{array}{cc} {\dot{J}}_{k}{\left(w\right)}_{DMEE} & =\sum_{l\in N_{k}}c_{lk}\frac{\partial V{\left(e_{l,i}\right)}^{T}}{\partial w}\dot{w}\\ & =\sum_{l\in N_{k}}{c_{lk}\left(‒\mu_{l,DMEE}\right)∥\frac{\partial V(e_{l,i})}{\partial w}∥}^{2}. \end{array}$$

The switching time is determined as

(50) $$\left|{\dot{J}}_{k}{\left(w\right)}_{DMEE‒SAS}\right|>\left|{\dot{J}}_{k}{\left(w\right)}_{DMEE}\right|\Leftrightarrow V\left(e_{l,i}\right)<V\left(0\right)‒\frac{1}{2}\sqrt{\frac{\mu_{l,DMEE}}{\mu_{l,DMEE‒SAS}}}\left(l\in N_{k}\right).$$

When the condition of Equation (50) is met, we should switch from the DMEE-SAS algorithm to the DMEE-SAS algorithm. We introduce the following auxiliary variable:

$$s_{k,i}=\left\{\begin{array}{c} 1,V\left(e_{k,i}\right)<V\left(0\right)‒\frac{1}{2}\sqrt{\frac{\mu_{k,DMEE}}{\mu_{k,DMEE‒SAS}}},\\ 0,\mathrm{otherwise}. \end{array}\right.$$

This yields the following algorithm, which we refer to as the improving DMEE-SAS algorithm:

(51) $$\left\{\begin{array}{c} \varphi_{k,i+1}=w_{k,i}‒s_{k,i}\mu_{{}_{k,DMEE‒SAS}}\frac{2}{\sigma^{2}L}\left[V\left(0\right)‒V\left(e_{k,i}\right)\right]\sum_{j=i‒L+1}^{i}G_{\sigma \sqrt{2}}\left(e_{k,i}‒e_{k,j}\right)\left(e_{k,i}‒e_{k,j}\right)\\ \;\;\;\;\left(u_{k,j}‒u_{k,i}\right)‒\left(1‒s_{k,i}\right)\mu_{{}_{k,DMEE}}\frac{1}{\sigma^{2}L}\sum_{j=i‒L+1}^{i}G_{\sigma \sqrt{2}}\left(e_{k,i}‒e_{k,j}\right)\left(e_{k,i}‒e_{k,j}\right)\left(u_{k,j}‒u_{k,i}\right)\\ w_{k,i+1}=\sum_{l\in N_{k}}c_{lk}\varphi_{l,i+1}. \end{array}\right.$$

For the purpose of clarity, we summarize the procedure of the Improving DMEE-SAS algorithm in Algorithm 2.
**Algorithm 2:** Improving DMEE-SAS Algorithm **Initialize:** $w_{k,i}=0$  **for**
i=1:T **for each node**
*k*:  **Adaptation**  **each node calculates the switching time using Equation (50).**  **each node updates intermediate estimate**
$\varphi_{k,i}$
**according to the first equation of Equation (51).**  **Combination**  $w_{k,i+1}=\sum_{l\in N_{k}}c_{lk}\varphi_{l,i+1}$ **end for**

## 4. Simulation Results

**Figure 1:**
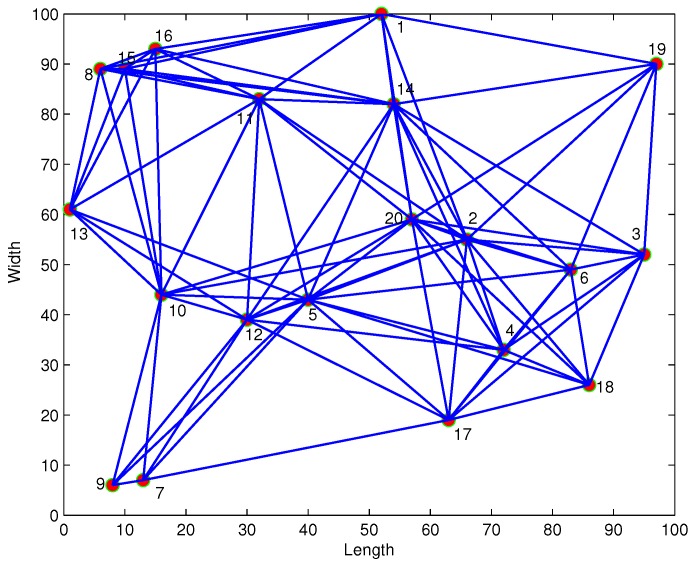
Network topology.

Twenty sensors are randomly placed in a square 100×100 shown in Figure 1. The communication distance is set as 50. In this paper, the performance of the steady-state network MSD [12] is adopted for performance comparison. All of the performance measures are averaged over 100 trials.

We employ the super-Gaussian distribution as the noise model in our simulations. We generate the noise from the zero-mean generalized Gaussian distribution of probability density function $q_{V}\left(v\right)=\propto \exp\left(‒{\left|v\right|}^{p}\right)$, where *p* is a positive shape parameter of probability density function [50]. We set p=0.6 to make the noise distribution be super-Gaussian.

(a) In Stationary Environment

Here, the proposed DMEE-SAS algorithm performance is compared with that of some existing algorithms in the literature. We assume the communication link is the ideal link. The unknown parameter vector $w^{0}$ is set to ${\left[\frac{1}{\sqrt{6}},\frac{1}{\sqrt{6}},\frac{1}{\sqrt{6}},\frac{1}{\sqrt{6}},\frac{1}{\sqrt{6}},\frac{1}{\sqrt{6}}\right]}^{T}$.

**Figure 2:**
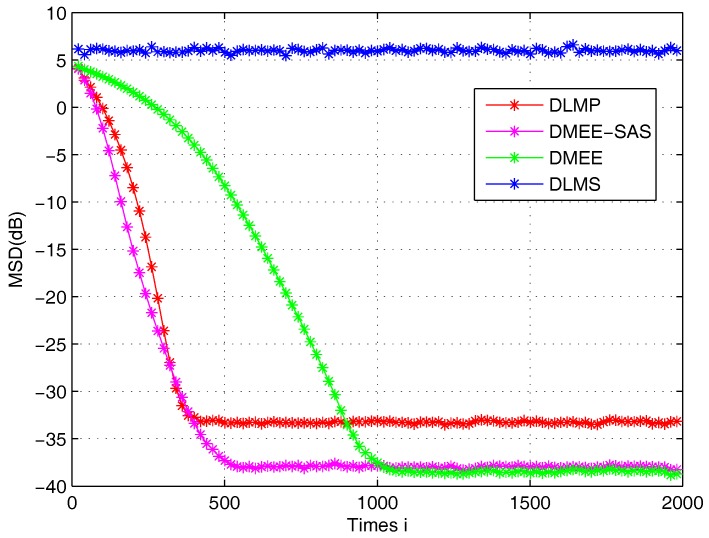
Transient mean-square-error (MSD) curves.

We set the window length L=8 and kernel size σ=1.5 for both DMEE and DMEE-SAS algorithms. Furthermore, the *p* is 1.2 for the DLMP algorithm. The steady state MSD curves are plotted in Figure 2. It is found that the DMEE-SAS algorithm is robust to the non-Gaussian noises and performs better than the DLMP algorithm [26] and DLMS [12]. The DMEE-SAS algorithm achieves a better convergence performance than the DMEE [31] algorithm when the DMEE-SAS and DMEE algorithms achieve comparable performance.

(b) In Non-stationary Environment

Here, the simulations are carried out in the same environments as those shown in stationary environment, except for the optimal estimator $w^{0}$. We compare the proposed Improving DMEE-SAS algorithm with other algorithms.

Motivated by [51], we assume a time-varying $w^{0}$ of length 6 as follows:

$$w_{i}^{0}=\frac{1}{2}{\left[a_{1,i},a_{2,i},a_{3,i},a_{4,i},a_{5,i},a_{6,i}\right]}^{T},$$

where $a_{k,i}=\left[\cos\left(wi+\frac{\left(k‒1\right)}{2}\pi \right)\right]$ for k=1,2,3,4,5,6 and $w=\frac{\pi }{3000}$.

**Figure 3:**
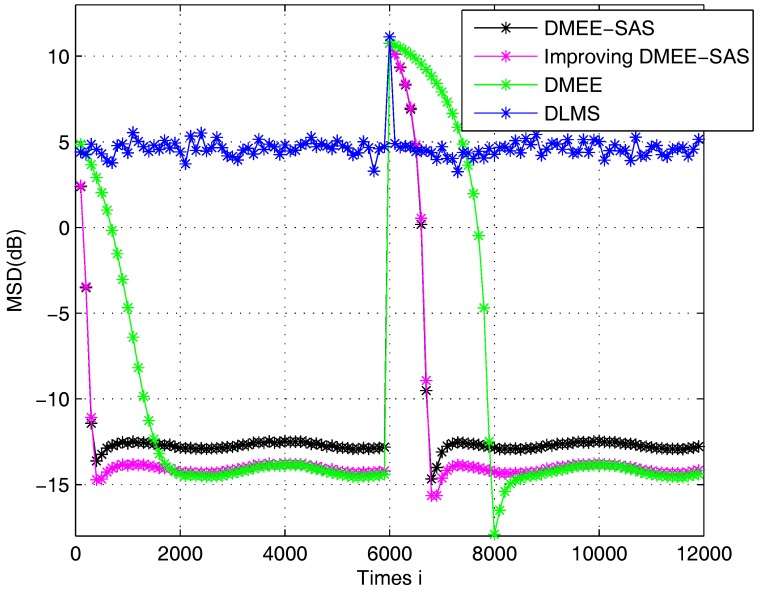
MSD learning curves in a non-stationary environment.

The unknown link is assumed to change at time 6000. In Figure 3, the Improving DMEE-SAS algorithm can detect the weight vector change and the performance of it is better than the DLMS algorithm. We observe that Improving DMEE-SAS and DMEE algorithms achieve comparable performance and Improving DMEE-SAS achieves better convergence performance than the DMEE algorithm. When compared with the DMEE-SAS algorithm, the Improving DMEE-SAS algorithm exhibits a significant improvement in performance when the estimate is close to the optimal estimator. The Improving DMEE-SAS algorithm achieves a low MSD and fast rate of convergence in the non-stationary environment.

## 5. Conclusions

In this paper, a robust diffusion estimation algorithm with self-adjusting step-size is developed which is called the DMEE-SAS algorithm. The mean and mean square convergence analysis of this new algorithm are carried out, and a sufficient condition for ensuring the stability is obtained. Simulation results illustrate that the DMEE-SAS algorithm can achieve better performance than the DLMS, robust DLMP, and DMEE algorithms in non-Gaussian noise scenario. In addition, we propose the Improving DMEE-SAS algorithm, using it in the non-stationary scenario where the unknown parameter is changing over time. The Improving DMEE-SAS algorithm combined the DMEE-SAS algorithm with the DMEE algorithm, and it can avoid the small effective step-size of the DMEE-SAS algorithm when close to the optimal estimator.
